# Supplementary material for: Moisturizing and Antioxidant Effects of Artemisia argyi Essence Liquid in HaCaT Keratinocytes
Source: Int J Mol Sci. 2023 Apr 6;24(7):6809. doi: 10.3390/ijms24076809 (PMC10095007; doi:10.3390/ijms24076809)
Supplement: Supplementary file 1 [file ijms-24-06809-s001.zip › ijms-2262067-supplementary.pdf]

# *Supplementary Material*

**Supplementary Table S1. The content of flavonoids, polysaccharides, phenols in AL.**

| constituents | Content(mg/mL) |
|---|---|
| Flavonoids (apigenin) | 0.2 ± 0.02 |
| Terpene (oleanolic acid) | 9.33 ± 0.59 |
| Polysaccharides (glucose) | 1.02 ± 0.02 |
| polyphenols (gallate) | 0.31 ± 0.00 |

**Supplementary Table S2.Gene-specific primers.**

| Gene | Forward (5’ to 3’) | Reverse(5’ to 3’) |
|---|---|---|
| AQP3 | GGGGAGATGCTCCACATCC | AAAGGCCAGGTTGATGGTGAG |
| HAS2 | CTCTTTTGGACTGTATGGTGCC | AGGGTAGGTTAGCCTTTTCACA |
| HAS3 | CAGCCTATGTGACGGGCTAC | CCTCCTGGTATGCGGCAAT |
| TGM-1 | GCACCACACAGACGAGTATGA | GGTGATGCGATCAGAGGATTC |
| TJP-1 | ACCAGTAAGTCGTCCTGATCC | TCGGCCAAATCTTCTCACTCC |
| SOD2 | CTGCTGGGGATTGATGTGTGG | TGCAAGCCATGTATCTTTCAGT |
| FLG | GGACAGGAACAATCATCGGGG | CAACCTCTCGGAGTCGTCTG |

**Supplementary Table S2 (continued)**

| Gene | Forward (5' to 3') | Reverse(5' to 3') |
| --- | --- | --- |
| GPX1 | AGTCGGTGTATGCCTTCTCGG | GCAGCTCGTTCATCTGGGTGTA |
| Nrf2 | TCAGCGACGGAAAGAGTATGA | CCACTGGTTTCTGACTGGATGT |
| HO-1 | AAGACTGCGTTCCTGCTCAAC | AAAGCCCTACAGCAACTGTCG |
| β-actin | AAATCGTGCGTGACATCAAAGA | GCCATCTCCTGCTCGAAGTC |
